# Long Term Successful Weight Loss with a Combination Biphasic Ketogenic Mediterranean Diet and Mediterranean Diet Maintenance Protocol

**DOI:** 10.3390/nu5125205

**Published:** 2013-12-18

**Authors:** Antonio Paoli, Antonino Bianco, Keith A Grimaldi, Alessandra Lodi, Gerardo Bosco

**Affiliations:** 1 Department of Biomedical Sciences, University of Padova, Padova 35131, Italy; E-Mails: alessandra.lodi@studenti.unipd.it (A.L.); gerardo.bosco@unipd.it (G.B.); 2 Sport and Exercise Sciences Research Unit, University of Palermo, Palermo 90146, Italy; E-Mail: antonino.bianco@unipa.it; 3 Biomedical Engineering Laboratory, Institute of Communication and Computer Systems, National Technical University of Athens, Athens 15773, Greece; E-Mail: keith.grimaldi@gmail.com

**Keywords:** ketogenic diet, Mediterranean diet, long term, phytoextracts

## Abstract

Weight loss protocols can only be considered successful if they deliver consistent results over the long term—a goal which is often elusive, so much so that the term “yo-yo” is used to describe the perennial weight loss/weight regain battle common in obesity. We hypothesized that a ketogenic Mediterranean diet with phytoextracts (KEMEPHY) combined with the acknowledged health benefits of traditional Mediterranean nutrition may favor long term weight loss. We analysed 89 male and female obese subjects, aged between 25 and 65 years who were overall healthy apart from being overweight. The subjects followed a staged diet protocol over a period of 12 months: 20 day of KEMEPHY; 20 days low carb-non ketogenic; 4 months Mediterranean normocaloric nutrition; a second 20 day ketogenic phase followed by 6 months of Mediterranean normocaloric nutrition. For the majority of subjects (88.25%) there was significant loss of weight (from 100.7 ± 16.54 to 84.59 ± 9.71 kg; BMI from 35.42 ± 4.11 to 30.27 ± 3.58) and body fat (form 43.44% ± 6.34% to 33.63% ± 7.6%) during both ketogenic phases followed by successful maintenance, without weight regain, during the 6 month stabilization phase with only 8 subjects failing to comply. There were also significant and stable decreases in total cholesterol, LDLc, triglycerides and glucose levels over the 12 month study period. HDLc showed small increases after the ketogenic phases but over the full 12 months there was no significant change. No significant changes were observed in ALT, AST, Creatinine or BUN. The combination of a biphasic KEMEPHY diet separated by longer periods of maintenance nutrition, based on the traditional Mediterranean diet, led to successful long term weight loss and improvements in health risk factors in a majority of subjects; compliance was very high which was a key determinant of the results seen.

## 1. Introduction

Health organizations report a worldwide increased prevalence of overweight and obesity [1] which is a great source of concern considering the fact that obesity and in particular abdominal obesity is one of the principle risk factors for cardiovascular disease and is strongly linked to dyslipidaemia, hypertension, diabetes and metabolic syndrome [2].

The most commonly accepted weight loss strategy is based on a simple reduction of daily calorie intake as part of a low fat/high carbohydrate diet but there are still no clear data about which dietary protocols are most effective in both the short and long term [3] or even what is the correct nutritional approach in general [4]. There has been increased interest in recent years in very low carbohydrate ketogenic diets (VLCKD) [5] that have undoubtedly been shown to be effective, at least in the short to medium term [3], as a tool to tackle obesity, hyperlipidemia and some cardiovascular risk factors [5]. Ketogenic diets are characterized by a reduction in carbohydrates (usually to less than 50 g/day) and a relative increase in the proportions of protein and fat [6]. After a few days of such a nutritional regimen there is an increase of the “so called” ketone bodies that can be used by tissues for energy as an alternative to glucose. It is important to underline that this kind of mild ketosis should not be confused with the pathological ketosis of diabetes, indeed to reinforce this difference Hans Krebs called it “physiological ketosis” [7]. While there are many studies which demonstrate that a ketogenic diet, at least in the short-term, results in greater weight loss than low-fat diets [8], from a long term perspective the success of a nutritional approach is defined by the amount of weight regain [9] and, from this point of view, fewer data are available [10], in particular regarding so-called weight cycling or “yo-yo” effect [11,12]. Recently Sumitharn and colleagues have demonstrated that the increases in circulating ghrelin and in subjective appetite which accompanied a hypocaloric diet were reduced with a ketogenic approach [10]. Thus, we hypothesized that certain aspects of the ketogenic diet such as muscle mass retention, RMR (resting metabolic rate) and orexigenic hormone stability combined with the acknowledged health benefits of traditional Mediterranean nutrition may favor long term weight loss. The aim of our study was to investigate the effect on weight and body composition of two short periods of a modified ketogenic diet, *i.e.*, a very low carbohydrate ketogenic diet with phytoextracts (KEMEPHY) [13,14,15] interspersed between longer periods of maintenance nutrition, based on the traditional Mediterranean diet, over a total period of 12 months in obese/overweight healthy subjects.

## 2. Subjects and Methods

The present study was designed as a retrospective analysis of the medical records of patients followed by our private nutritional service held in three fitness and “weight control” centers in Veneto and Emilia Romagna regions (Italy) between 2006 and 2010. Patient charts were examined from the first clinical evaluation until after one year of dietary therapy supervised by a healthcare professional. The exclusion criteria from the baseline sample included: endocrine disease and cancer, which might induce weight variation, and severe mental illness. Inclusion criteria was BMI > 30, age between 25 and 65 years. Of 327 patients analyzed, 89 obese subjects were selected that underwent a ketogenic Mediterranean diet with phytoextract (KEMEPHY), of these 81 fulfilled our inclusion criteria for this retrospective analysis: no use of antidepressant drugs, no diabetes and no change in quantity and quality of physical activity during the analyzed time period. Of these 81 subjects, 68 completed the one year follow up protocol (84%). The general characteristics of the 68 remaining subjects analyzed were: age 49.17 ± 10 years, height 167 ± 10 cm, weight 100.67 ± 16.54 kg, BMI 35.82 ± 4.11. All subjects were Caucasian (59 males, 12 females). During the first medical visit (*t*0) subjects were educated on the KEMEPHY protocol and underwent anthropometric measurement, body composition and blood analysis. The year of treatment involved:

- An initial 20 days of very low carbohydrate ketogenic diet (K1)
- Followed by 20 days of a low carbohydrate non ketogenic diet (stabilization) (LC1)
- A first period of 4 months of normal caloric Mediterranean diet (M1)
- A second 20 day very low carbohydrate ketogenic diet (K2)
- 20 Days of low carbohydrate non ketogenic diet (LC2)
- Final 6 months of normal caloric Mediterranean diet (M2)

**Figure 1:**
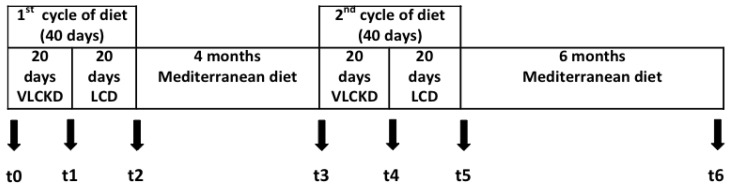
Experimental design *t*0 = start (medical examination, anthropometric and body composition analysis, blood analysis); *t*1 = end of K1 (medical examination, anthropometric and body composition analysis); *t*2 = end of LC1 (medical examination, anthropometric and body composition analysis, blood analysis); *t*3 = start of K2 (medical examination, anthropometric and body composition analysis); *t*4 = end of K2 (medical examination, anthropometric and body composition analysis); *t*5 = end of LC2 (medical examination, anthropometric and body composition analysis); *t*6 = one year recall (medical examination, anthropometric and body composition analysis, blood analysis). K = very low carbohydrate ketogenic diet, LC = low carbohydrate diet.

During the KEMEPHY period the subjects followed a commercially available protocol called TISANOREICA^®^ [13,14,15]. Two weight loss intervention periods were used as previously available clinical data (unpublished) suggested that this was required to achieve a 10% weight loss. Subjects were analyzed at seven time points: before starting the diet (*t*0), after K1 (*t*1), after LC1 (*t*2), after M1 (*t*3), after K2 (*t*4), after LC2 (*t*5) and after M2 *i.e.*, approximately one year after first visit (*t*6)—see Figure 1. All subjects gave their informed consent to data use and the study was approved by the Ethical Commission of the Department of Biomedical Sciences of the University of Padova. Efforts to maximize retention in the protocol included e-mail and telephone reminders for appointments and a weekly phone call to verify compliance.

### 2.1. Diet Protocols

The diets were explained to all subjects by a qualified dietician during an individual visit. Dietary intake was measured with a validated 3-day food diary [16] and analyzed by Dietnext^®^ software (Caldogno, Vicenza, Italy). In the KEMEPHY protocol subjects almost totally exclude carbohydrates during the first three weeks. A detailed menu containing permitted and non-permitted foods was provided to each participant, along with the components of the ketogenic Mediterranean with phytoextracts diet. The diet consumed was primarily made of beef & veal, poultry, fish, raw and cooked green vegetables without restriction, cold cuts (dried beef, carpaccio and cured ham), eggs and seasoned cheese (e.g., parmesan). The drinks allowed were infusion tea, moka coffee and herbal extracts. The foods and drinks that subjects avoided included alcohol, bread, pasta, rice, milk, yogurt, soluble tea and barley coffee. In addition to facilitate the adhesion to the nutritional regime, each subject was given a variety of specialty meals constituted principally of protein and fibers. These meals (TISANOREICA^®^, Asigliano Veneto, Vicenza, Italy) that are composed of a protein blend obtained from soya, peas, oats (equivalent to 18 g/portion) and virtually zero carbohydrate (but that mimic their taste) were included in the standard ration [14]. During the KEMEPHY protocol, the subjects also consumed some specific herbal extracts. Briefly subjects consumed 20 mL of four different herbal extracts (for a total amount of 80 mL) the objective being to: ameliorate the commonly reported symptoms of weakness and tiredness during the ketosis, improve glycaemic control and increase bile secretion helping digestion (choleretic effect) [13,14] (herbal blends are described in detail elsewhere) [14]. During the ketogenic diet periods, subjects assumed 1 caplet in of a multivitamin-mineral supplement each morning [17,18,19]. The composition of the caplets (Multivitaminico Balestra e Mech, Gianluca MechSpA, Asigliano Veneto, Vicenza, Italy) has been detailed previously [14].

During the months of the Mediterranean diet, subjects were instructed to follow a typical diet composed mainly of, whole grains (bread, pasta, whole wheat, rice), potatoes, meat, fish, eggs, poultry, vegetables, legumes, fruits, condiments (mainly olive oil), whole milk and wine. During the Mediterranean diet period the average macronutrients distribution was: 58% carbohydrate, 15% protein and 27% lipids (Kcal 1800 ± 108); the main sources of added fat were 30 to 50 g of olive oil per day [20]. During the ketogenic period the prescribed daily intake of carbohydrate was about 30 g per day and the energy distribution of daily macronutrients was 12% carbohydrate, 36% protein and 52% lipids (Kcal 976 ± 118). During the low carbohydrate period the distribution was 25% carbohydrate, 31% protein and 44% lipids (Kcal 1111 ± 65) (Table 1).

**Table 1:** Characteristics of diets (data are expressed as mean and SD).

Macronutrients	Ketogenic Phase	Lowcarbohydrates Phase	Mediterranean Phase
Kcal/day	976 ± 118	1111 ± 65	1800 ± 248
Protein (% totaldaily Kcal)	41 ± 2	27 ± 2	15 ± 2
Fat (% totaldaily Kcal)	46 ± 4	41 ± 2	27 ± 3
Carbohydrate (% totaldaily Kcal)	12 ± 2	33 ± 2	58 ± 4
Protein (g/day)	100 ± 11	74 ± 11	67.5 ± 9
Fat (g/day)	51 ± 9	50 ± 2	54 ± 6
Carbohydrates (g/day)	30 ± 0.2	91 ± 5	261 ± 18

### 2.2. Analysis

Dietary intake was measured by validated 3-day food diary [16,21] and analyzed by Dietnext^®^ (Caldogno, Vicenza, Italy) software. At each visit (timepoint *t*1–*t*6) subjects underwent anthropometric measurement, blood pressure measurements and body composition analysis, the latter was assessed using bioelectrical impedance analysis (BIA Akern Bioresearch, Pontassieve, FI, Italy) which is a non-invasive and portable method for the estimation of fluid compartments, fat and fat-free mass in healthy subjects. Bioelectrical impedance analysis was chosen because it is a reliable method and its safety, convenience and non-invasive nature makes it useful procedure to be deployed in the routine monitoring of body composition also during the ketogenic diet [22,23]. At *t*0, *t*3, and *t*6 fasting venous blood samples were collected at weeks 0 and 6 for total cholesterol (CHOL-T), triacylglycerol (TG), high-density lipoprotein cholesterol (HDL-C), low-density lipoprotein cholesterol (LDL-C), glucose, blood urea nitrogen (BUN), uricemia, eythrocyte sedimentation rate (ESR), creatinine, alanine transaminase (ALT), aspartate transaminase (AST), gamma-glutamyl transpeptidase (GGT). Blood was collected in EDTA treated vacutainer tubes. All measurements were made on a ROCHE/HITACHI 912, (Roche Diagnostics Ltd., Basel, Switzerland). Fasting total cholesterol was measured with an enzymatic colorimetric method. HDL-C and LDL-C were measured by an enzymatic colorimetric test in homogenous phase. Triglycerides with an enzymatic colorimetric TRINDER modified final point method. Plasma glucose was determined with the plasma glucose GOD-PAP enzymatic colorimetric method. Plasma urea nitrogen was measured using UV kinetic test. ALT and AST with IFCC enzymatic kinetic method. GGT with the SZASZ (kinetic photometric method), colorimetric method. Creatinine was measured using the JAFFE method with compensation, kinetic colorimetric test, and uric acid was determined using an enzymatic colorimetric method.

### 2.3. Statistical Analysis

The effect of the diet intervention was assessed using one way repeated-measures ANOVA. For body weight and fat percentage the measurements at *t*0, *t*1, *t*2, *t*3, *t*4, *t*5 and *t*6 were considered. For blood variables only *t*0, *t*3 and *t*6 time points were taken into account. When significant effects were found, post hoc analysis was performed using Tukey’s test. An alpha level of *p* < 0.05 was used to denote a significant effect. Kolmogorov-Smirnov tests were used to assess the normality of the data. Mauchley’s test of sphericity assessed the homogeneity of variance for the data. All statistical analyses were performed using the software package GraphPad Prism version 6.00 for Mac [24]. Values are represented as means and standard deviation (SD).

## 3. Results

**Figure 2:**
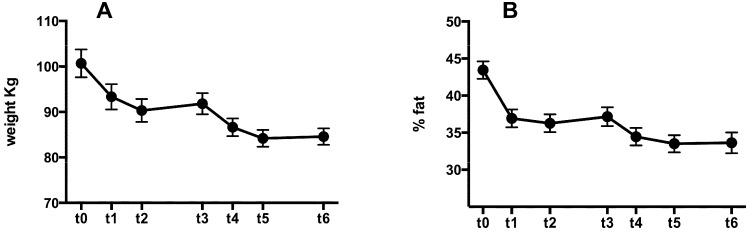
Changes in body weight (**A**) and fat percentage (**B**) from baseline to month 12. Error bars indicate standard error of the mean.

There was a significant decrease in body weight after the first ketogenic period (*p* < 0.0001 *t*0 *vs*. *t*1); there was no significant difference between *t*1 and *t*2 nor between *t*2 and *t*3. At *t*4 body weight was significantly decreased compared to *t*3 and *t*1 (*t* < 0.01 and *t* < 0.001 respectively) after which it stabilized with no further significant changes at *t*5 and *t*6 (see Figure 2A and Table 2). The same pattern was seen also for body fat percentage (see Figure 2B and Table 2). Comparing bodyweight and body fat at *t*6 with *t*0 revealed that after one year there was an overall significant decrease in both parameters with no signs of weight regain over the course of the study. Systolic blood pressure showed a significant decrease comparing *t*0 *vs*. *t*2 (from 125 ± 10 to 117 ± 6 *p* < 0.01) whilst there was a decrease albeit not significant of diastolic blood pressure (from 86 ± 5 to 82 ± 8).

**Table 2:** Changes in body weight and body fat percentage during one year diet protocol. Values are expressed as mean and standard deviation. Significance was reported in Results section.

Anthropometric Data	*t*0	*t*1	*t*2	*t*3	*t*4	*t*5	*t*6
Body weight	100.7 ± 16.54	93.34 ± 15.04	90.33 ± 13.57	91.81 ± 12.58	86.64 ± 10.56	84.2 ± 10.04	84.59 ± 9.71
% Body fat	43.44 ± 6.34	36.93 ± 6.49	36.26 ± 6.46	37.15 ± 6.82	34.46 ± 6.3	33.50 ± 6.18	33.63 ± 7.6

Blood measurements (Table 3) revealed a significant decrease in total cholesterol at *t*2 (*p* < 0.0001) *vs*. *t*0, followed by non-significant differences between *t*2 and *t*6 and a significant decrease comparing *t*6 with *t*0 (*p* = 0.0003). HDL-C showed a significant increase after the ketogenic and low-carbohydrate phases (K1 and LC1; at *t*2) but after 1 year there was overall no significant difference compared to starting (*t*6 *vs*. *t*0), the increase seen was short term. LDL-C also decreased at *t*2 and in contrast to HDL the decrease remained significant throughout the study (*p* < 0.0001 *t*2 *vs*. *t*0; *p* = 0.004 *t*6 *vs*. *t*0). TG declined significantly at *t*2 compared to *t*0 (*p* = 0.0006) and for *t*6 *vs*. *t*0 (*p* = 0.01) although there was no significant difference between *t*2 and *t*6. Finally blood glucose decreased significantly at *t*2 *vs*. *t*0 (*p* < 0.0001) after which it rose but at the end of the 12 months it was still significantly lower at *t*6 *vs*. *t*0 (*p* = 0.0004). No significant changes were observed in ALT, AST, GGT, Creatinine or BUN.

**Table 3:** Changes in blood biochemical and pressure parameters at baseline (*t*0), after first period of ketogenic diet and low carbohydrate diet (*t*2) and after one year from the start (*t*6). Values are expressed as mean and standard deviation.

Blood Parameters	*t*0	*t*2	*t*6	*t*0 *vs*. *t*2	*t*0 *vs*. *t*6	*t*2 *vs*. *t*6
Chol-tot	193.2 ± 37.87	171.9 ± 31.94	179.8 ± 32.42	*p* < 0.0001	*p* = 0.0003	n.s.
HDL-C	43.03 ± 6.09	49.59 ± 8	44.59 ± 8	*p* < 0.0001	n.s.	*p* < 0.001
LDL-C	144.5 ± 58.4	108.0 ± 42.66	122.9 ± 42.25	*p* < 0.0001	*p* = 0.0004	*p* < 0.0001
TG	112.7± 61.02	88.62 ± 40.65	95.45 ± 39.99	*p* = 0.0006	*p* = 0.0106	n.s.
Glu	102.6 ± 11.5	90.31 ± 8.45	95.31 ± 8.45	*p* < 0.0001	*p* = 0.0004	*p* < 0.0001
ALT	18.75 ± 11.6	16.53 ± 6.72	17.11 ± 9.3	n.s.	n.s.	n.s.
AST	18 ± 8.69	17.13 ± 7.2	17.76 ± 5.43	n.s.	n.s.	n.s.
GGT	20.68 ± 16.16	16.1 ± 5.3	17.8 ± 6.8	*p* = 0.012	*p* < 0.05	n.s.
Creatinine	0.79 ± 0.16	0.76 ± 0.07	0.77 ± 0.1	n.s.	n.s.	n.s.
BUN	15.87 ± 3.83	16.1 ± 85.29	15 ± 3.87	n.s.	n.s.	n.s.
Uric acid	4.56 ± 0.86	4.2 ± 0.64	4.01 ± 0.91	*p* < 0.01	*p* < 0.05	n.s.
SBP	125 ± 10	117 ± 6	118 ± 4	*p* < 0.01	*p* < 0.01	n.s.
DBP	86 ± 5	82 ± 8	82 ± 5	n.s.	n.s.	n.s.

n.s. = not significant.

## 4. Discussion

There is a growing interest in the scientific community and in the public media in very low carbohydrate ketogenic diets (VLCKD) and there are good reasons for this; the majority of randomized controlled trials comparing *ad libitum* ketogenic low-carbohydrate diets with low-fat diets have found greater weight loss over six months in the former [3,20,25]. A recent metanalysis reported that subjects following a VLCKD achieved significantly greater long-term reductions in body weight [8].

**Figure 3:**
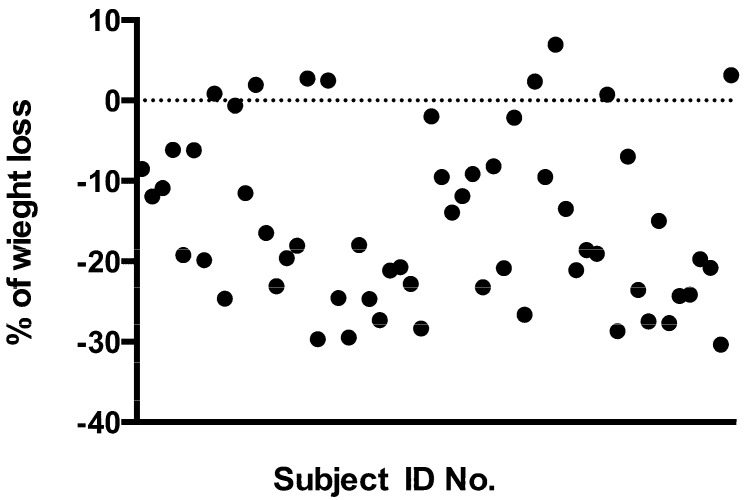
Changes in weight (% of change) of each subject (*t*6 compared to *t*0). Basal value is represented by the line zero. Each circle represents a single subject.

One of the most common critiques raised against the use of VLCKD is the so-called “yo-yo” effect, *i.e.*, the weight regain cycle [11,12,26]. In other words some opponents and doubters of VLCKD suggest that any beneficial effects are only transient. There is no universally accepted definition of “successful weight loss maintenance” following a diet but a reasonable candidate would be that proposed by Wing and Hill in 2001, which defines it as “individuals who have intentionally lost at least 10% of their body weight and kept it off at least one year” [27]. The criterion of 10% is chosen for its well documented effects in the improvements in risk factors for diabetes and cardiovascular disease, while the one year duration criterion was proposed in agreement with the USA Institute of Medicine [28]. The data from our present study suggest that two brief periods of a “Mediterranean” variant on the VLCKD theme (which we call KEMEPHY) are able to induce significant weight and body fat loss that was maintained for at least one year. In particular the weight loss reached at six months, after the second cycle of VLCKD, was maintained, without weight regain, over the subsequent six months of normocaloric Mediterranean nutrition. The mechanisms underlying the effects of VLCKD on weight loss is still a subject of debate. One hypothesis is that the use of energy from protein in VLCKD is an “expensive” process for the body and so can lead to a “waste of calories” and therefore increased weight loss compared to other “less expensive” diets [29]. During the first phase of a VLCKD 60–65 g of glucose per day are needed by the body, 16% of this is obtained from glycerol whilst the major part derived via gluconeogenesis from proteins, of either dietary or tissue origin [30]. Gluconeogenesis is an energy-demanding process calculated at approximately 400–600 Kcal/day (due to both endogenous and food source proteins [29]. There is however no direct experimental evidence to support this intriguing hypothesis, on the contrary a recent study reported that there were no changes in resting energy expenditure after a VLCKD [31]. Some authors claim instead that the results obtained with ketogenic diets could be attributed to a reduction in appetite due to higher satiety effect of proteins [29,32] or to some effects on appetite control hormones [10]. Other authors suggest a possible direct appetite suppressant action of the ketone bodies [33]. But regardless the mechanisms involved in the weight loss effects of KD, there is substantial agreement about its medium term efficacy. Nevertheless, as stated before, one of the major problems in weight control is the prevention of weight regain. In the present study the majority of subjects maintained >10% weight loss at 12 months but we detected 8 subjects in which the weight loss was not maintained at all (Figure 3); these subjects were included in the final statistical calculations but the post dietary analysis showed that they were not compliant with nutritional guidelines given for the Mediterranean diet period. These subjects returned to their previous nutritional habits (“junk” food, high glycaemic index, *etc*.) with a mean “real” daily intake of 2470 Kcal rather than the prescribed 1800 Kcal. Hence the compliance to this one year protocol was 88.25% in accordance with previous data [14]. Beyond the most obvious cause of weight regain, *i.e.*, a return to previous “unhealthy” habits, the physiological basis of weight regain appears to be complex [12,26,34]. It is reasoned that the maintenance of weight loss is difficult due to many factors including reduced resting metabolic rate (RMR), insulin & leptin resistance and changes in the levels of several hormones involved in the homeostatic regulation of body weight [35]. RMR could be affected by the loss of muscle mass due to an inadequate protein intake during dieting. The suggested daily protein consumption is around 15% in a classical hypocaloric Western diet of about 1200 Kcal/day so the actual protein content will be approximately 45 g (180 Kcal; 4 Kcal/g). Hypothesizing a body weight of 70 kg this daily protein intake will be 0.64 g per kilogram of body weight which is a possible cause of muscle loss and consequent reduction of resting energy expenditure. The VLCKD on the other hand appears not to influence (either positively or negatively) the basal energy expenditure, but recent data showed that it could improve fat oxidation and therefore lower the respiratory ratio [31,36]. Regarding hormonal influences on weight regain a possible explanation involves a long-term increase in orexigenic signals. Sumithran and co-workers showed that a very low calorie diet causes a persistent elevation of the circulating mediators of appetite that encourages weight regain even one year after initial weight reduction [35]. On the other hand it has been demonstrated that a ketogenic diet has only a minor effect on ghrelin levels and that the subjective ratings of appetite were lower when participants were in a state of physiological ketosis [10].

We have to consider that there are some concerns among physicians and nutritionists about various aspects of VLCKD [37]. The main proposed risk of VLCKD is possible kidney damage due to high levels of nitrogen excretion during protein metabolism which can cause an increase in glomerular pressure and hyper-filtration [30]. But there are conflicting results: some author suggest the possibility of renal damage [38,39] based on results from animal studies whilst others, looking at both animal models, meta-analyses and human studies suggest, on the contrary, that even high levels of protein in the diet do not damage renal function [40,41]. In subjects with intact renal function higher dietary protein levels have been reported to cause some functional and morphological adaptations but without negative effects [42]. Given the inconsistency of the evidence, a cautious approach is warranted in subjects with renal insufficiency, including sub-clinical, and with kidney transplant patients. Although it should also be considered that VLCKD is not necessarily a “high protein diet” (it may be higher in proportion, but not in actual content). Moreover recent research suggests that ketogenic diets may even cause a regression of diabetic nephropathy in mice [43] and that a VLCKD had no negative effects on renal function in Type 2 Diabetics. With regards to possible acidosis during VLCKD, since the concentration of ketone bodies never rises above 8 mmol/L [44] this risk is virtually nonexistent in subjects with normal insulin function. On the other hand there is a large amount of evidence for the benefits of Mediterranean nutritional approach on many health related outcomes. The Mediterranean diet is associated with a longer life span [45], lower rates of coronary heart disease [4], hypercholesterolemia [46], hypertension, diabetes and obesity [47]. But is difficult to isolate the “healthy” constituents of the Mediterranean diet, since it is not a single entity and varies between regions and countries. All things considered there is no “one size fits all” dietary recommendation and for this reason we have tried to merge the benefits of these two approaches: the long term “all-life” Mediterranean diet coupled with brief periods of a metabolism enhancing ketogenic diet.

## 5. Conclusions

In summary the data from this study demonstrate that the majority of subjects showed significant weight loss (10%) as a result of a two-phase VLCKD and were compliant both during the six month weight loss phase and the six month normocaloric maintenance phase, with no weight regain. We can suggest that the proposed protocol was generally successful because of (a) the protein mass protective effects of a VLCKD and (b) the prescription of a traditional Mediterranean diet in the post weight-loss phase was especially important for achieving “weight loss success”, *i.e.*, continued weight loss for at least one year.
